# Dietary Energy and Protein Levels Influence the Mutton Quality and Metabolomic Profile of the Yunshang Black Goat

**DOI:** 10.3390/foods13142271

**Published:** 2024-07-18

**Authors:** Zijian Li, Yanting Jiang, Muhammad Khan, Bai Xue, Xiaoqi Zhao, Binlong Fu, Weijuan Li, Baiji Danzeng, Xiaojun Ni, Qingyong Shao, Yina Ouyang

**Affiliations:** 1 Yunnan Animal Sciences and Veterinary Institute, Kunming 650224, China; lzj01071217@163.com (Z.L.); jiangyanting-2007@163.com (Y.J.); khanbwn011@gmail.com (M.K.); zhaoxiaoqi2023@163.com (X.Z.); binlongfu@163.com (B.F.); ynxmsyxh@126.com (W.L.); danzeng1376@163.com (B.D.); nixiaojun123@126.com (X.N.); shaoqingyong@163.com (Q.S.); 2 Animal Nutrition Institute, Sichuan Agricultural University, Chengdu 611130, China; xuebai68@163.com

**Keywords:** Yunshang black goat, meat quality, *longissimus lumborum* muscle, GC-MS, LC-MS

## Abstract

This study aimed to evaluate the impact of dietary energy and protein levels on the meat quality and metabolomic profile of Yunshang black goats. For this, 80 Yunshang black goats (male, 6 months old, with a mean live body weight of 35.82 ± 2.79 kg) were used in a completely randomized design with a 2 × 2 factorial dietary arrangement. The dietary treatments were (1) high energy (9.74 MJ/kg) with high protein (12.99%) (HEHP), (2) high energy (9.76 MJ/kg) with low protein (10.01%) (HELP), (3) low energy (8.18 MJ/kg) with high protein (13.04%) (LEHP), and (4) low energy (8.14 MJ/kg) with low protein (10.05%) (LELP). The experiment lasted 64 days, including 14 days for dietary adaptation and a 50-day feeding trial. At the end of the experiment, four animals from each treatment were slaughtered to assess their meat quality and metabolomic profiles. The pH value was greater for the goats fed the LELP diet compared with the other treatments. The LEHP-fed group’s meat was brighter (L*) than that of the other three groups. The HEHP-fed group had considerably more tender meat (*p* < 0.05) compared with the LEHP-fed group. Moreover, 72 and 183 differentiated metabolites were detected in the *longissimus* muscle samples by using gas chromatography–mass spectrometry and liquid chromatography–tandem mass spectrometry, respectively. The hydropathy and volatilities of raw meat were different (*p* < 0.05), suggesting changes in the meat flavor because of the dietary treatments. Based on the results, it can be concluded that feeding a high-energy- and high-protein-containing diet improved the tenderness, flavor, and fatty acid contents of mutton.

## 1. Introduction

Yunnan Province has 12.76 million goats of several breeds, including the Yunshang black goat breed. However, ancient breeds have poor production and reproductive performance, suggesting that these breeds are not suitable for large-scale farming. These animals are characterized by poor growth rates, lower carcass yields, small litter sizes, and higher vulnerability to disease in the local community [1]. The Yunshang black goat is a recently developed goat breed (approved by China’s National Livestock and Poultry Genetic Resources Commission in 2019), obtained by crossing the Nubia (♂) and Yunling black (♀) goat breeds. This is a moderately sized breed with compact stature and meat which has excellent growth potential (adult males and females can attain live body weights of up to 76 and 57 kg, respectively) and reproduction efficiency (kidding rate ranging between 186 and 236%) with better adaptability to low- (less than 1000 m) as well as high-altitude (about 2000 m) areas [1].

It is well known that diet-associated factors such as diet presentation, ingredient sources and their inclusions, and enrichment of a single nutrient or blend of nutrients can influence the growth performance, health, and meat quality of animals [2,3]. Moreover, ruminants have a unique four-compartment (rumen, reticulum, omasum, and abomasum) stomach, which makes them different from monogastric animals, particularly regarding nutrient utilization and bioenergetics [4]. Dietary contents such as energy and proteins are degraded by ruminal microbes into short-chain fatty acids (SCFAs) and microbial proteins, respectively. It is well established that changes in dietary energy and protein contents have a direct influence on ruminal pH, the molar ratio of SCFAs, and the health of the animals [5,6]. For example, feeding them a high-grain diet results in a drop in ruminal pH and higher production of lactic acid in goats [7]. Thus, changes in the dietary composition, particularly regarding protein and energy, may affect the deposition of fatty acid-based compounds in muscle tissue, which contribute to its flavor.

Meat quality is primely assessed by using several parameters, including meat flavors. Meat flavors are complex arrays of volatile organic compounds (VOCs) and non-volatile compounds, which are mainly responsible for different aromas [8,9]. The aroma imparts the taste and sensory characteristics and, in this way, determines the consumer’s preference [10]. Among the sensory characteristics of meat, its color, tenderness, flavor, and juiciness are important attributes which determine the quality of meat cuts, and studies have shown that tenderness is the most important factor in the perception of meat quality [11]. Gas chromatography–mass spectrometry (GC-MS) is a highly sensitive and high-throughput analytical platform which has been proven to be a useful tool for non-targeted studies on primary metabolism in a variety of applications [12]. Liquid chromatography–mass spectrometry (LC-MS) has become increasingly popular as a platform for metabolomics studies due to its high throughput, soft ionization, and good metabolite coverage [13].

Recently, a trend has been observed: studies are using the abovementioned advanced methods to investigate the impact of diet-associated factors on meat quality parameters [14,15,16]. For example, Wang et al. [17] used non-targeted metabolomics analysis based on GC-MS and LC-MS to determine the effects of different feeding methods on the potential meat flavor and taste components of Tan sheep. Liu et al. [18] evaluated the improvement in the meat quality of chicken meat by medium-chain monoglycerides through metabolomics. However, the metabolic changes in the muscle metabolites in Yunshang black sheep after being fed different energy and protein diets are still unclear. Therefore, we hypothesized that dietary changes in energy and protein levels might influence meat quality and meat metabolomics. Metabolomics is used to rapidly screen small-molecule metabolites in tissues, fluids, and cells under specific conditions, and it has been used to identify fraud in the food industry [19]. The objective of the current study is to evaluate the impact of dietary energy and protein levels on the meat quality and metabolomic profile of Yunshang black goats.

## 2. Materials and Methods

### 2.1. Experimental Site, Research Design, Animal Management, and Sample Collection

The current experiment was conducted at the Yunnan Academy of Animal Husbandry and Veterinary Sciences research facility after approval from the ethical committee of Yunnan Academy of Animal Husbandry and Veterinary Sciences (201911004). In this study, 80 male Yunshang black goats (approximately 6 months old and with live body weights of 35.82 ± 2.79 kg) were adjusted to four dietary treatments in a completely randomized design with 2 × 2 factorial treatment arrangements. The diets were categorized as follows: (1) high energy (9.74 MJ/kg) with high protein (12.99%) (HEHP), (2) high energy (9.76 MJ/kg) with low protein (10.01%) (HELP), (3) low energy (8.18 MJ/kg) with high protein (13.04%) (LEHP), and (4) low energy (8.14 MJ/kg) with low protein (10.05%) (LELP). The dietary energy and protein levels were determined by following the guidelines of the nutrient requirements of small ruminants (NRC-2007). The ingredients included and their chemical composition on a dry basis of the dietary treatments are given in Table 1. Chemical analyses of feed ingredients and dietary treatments were performed to determine the dry matter, crude protein, neutral detergent fiber, and acid detergent fiber contents by following the established protocols by Horwitz [20], and Van Soest, Robertson [21], respectively. The total duration of the experiment was 64 days, including a 14-day dietary adaptation period followed by a 50-day feeding trial. During the experimental period, bucks were housed individually in pens (1.5 m × 2.0 m) and given free access to fresh water and feed. The feeding frequency was twice (08:00 and 17:00 h) a day with 5% refusal adjustment on a daily basis to ensure the ad libitum intake. After the feeding trial, bucks were transported in the early morning to the slaughtering facility and then kept off-fed (without water and feed) for 24 h, weighted (live body ranged from 39 to 42 kg), and electrically stunned by following the rules and regulations of animal welfare. Samples of *longissimus lumborum* muscle were collected by dissecting between the 8th and 13th ribs of the carcass and immediately stored in liquid nitrogen containers at −80 °C. In addition, the meat samples (*longissimus lumborum* muscle) were also collected for meat quality parameters [22]. The preserved meat samples were subjected to subsequent analysis within two days after their collection for metabolomics and meat quality.

### 2.2. Meat Quality

The meat pH was measured 45 min post-slaughter by using a portable pH meter (Testo-205; Berlin, Germany). The fresh meat samples were analyzed (5 readings per sample) by using a digital colorimeter (WR-18 model, Guangdong Threenh Technology, Guangzhou, China) for meat brightness (L*), redness (a*), and yellowness (b*). The colorimeter was operated with a closed cone at 90-degree angles, and automatically recorded readings were used to evaluate the color coordinates. The meat samples (approximately 90 g of each) were cooked at a constant temperature of 80 °C for 2 h in a water bath following the detailed methodology by Khan et al. [23]. The pre-cooking and post-cooking weights of the samples were used to calculate the cooking loss. The cooked samples were dried by using tissue paper, sliced into 3 cm thick strips (n = 3 strips/sample), and drilled by a 1.27 cm sampler; then, the shear value was measured (3 times/sample) by using a muscle tenderness meter (RH-N50) to estimate the tenderness. The drip losses were measured according to the detailed protocol by Honikel [24].

### 2.3. Gas Chromatography–Mass Spectrometry (GC-MS) Analysis

Mass spectrometry (HS-SPME-GC-MS) analyses were performed according to previously published protocols with minor modifications [25]. Briefly, the *longissimus lumborum* muscle samples were thawed at 4 °C, the fascia layer was removed, and the samples were sliced. A 10 g sample from each goat was mixed with 2.5 μL of internal standard solution (o-dichlorobenzene, 0.5 μg/mL, in methanol) and 2 g of sodium chloride in a sealed bottle before heating at 120 °C for 30 min. The pre-cooled samples were extracted in a water bath at a constant temperature of 60 °C for 30 min by using solid-phase microextraction technology to absorb volatile substances in the headspace of the respective bottle. The extraction head was then placed in GC-MS equipment for thermal desorption for 3 min. Separation was performed by using a quartz capillary column, DB-heavy WAX (30 m × 250 μm × 0.32 μm; Agilent Tech, Beijing, China), with helium (99.999%) as the carrier gas. The separation conditions were as follows: the initial temperature was 40 °C, rising to 120 °C at 5 °C/min for 2 min and then rising to 220 °C at 7 °C/min for the next 5 min. The flow rate was adjusted to 0.8 mL/min. The mass spectrum settings were as follows: the ion source was electron ionization (EI), with a temperature of 230 °C, an electron energy of 70 eV, a scanning range of 45–350 *m*/*z*, and a scanning frequency of 2.76 times per second.

### 2.4. Liquid Chromatography–Tandem Mass Spectrometry (LC-MS) Analysis

The samples’ pre-treatment and the LC-MS analysis were carried out by following the standard methods of the previous study [26]. After vacuuming the 500 μL sample, 100 μL of 75% methanol aqueous solution was added. The mixture was vortexed for 60 s, filtered through a 0.2 μm organic filter membrane, and then subjected to the LC-MS machine. The LC-MS machine utilized an ACQUITY UPLC HSS T3 column (1.8 μm, 2.1 mm × 100 mm). The mobile phase for positive ion modes consisted of 0.1% formic acid in water (A) and 0.1% formic acid in acetonitrile (B), while for negative ion modes, it consisted of 2 mmol/L ammonium acetate in water (A) and acetonitrile (B). The column temperature was set to 40 °C with a loading volume of 3 μL. The gradient elution time ranged from 0 to 1.5 min (95%), 2.5 to 14 min (90%), 12 to 22 min (60%), 22 to 25 min (5%), and 25 to 30 min (0%). Detection was performed by using an electron spray ionization (ESI) source with both positive and negative ion modes. The mass spectrometer’s conditions were as follows: atomizing pressure of 60 psi, air curtain gas pressure of 35 psi, auxiliary pressure of 60 psi, temperature of 650 °C, and spray voltage of 5000 V for positive ions or −4000 V for negative ions.

### 2.5. Bioinformatics Analysis (Metabolite Profiling)

Raw data from GC-MS and LC-MS reads were converted into mzXML file format by using the MSConvert tool in the Proteowizard package (v3.0.8789). Peak detection, filtering, and alignment were performed by using the RXCMS software package (3.19 version). The data were analyzed for principal component analysis (PCA), partial least squares discriminant analysis (PLS-DA), and orthogonal partial least squares discriminant analysis (OPLS-DA) by using vegan packages in RStudio 3.6.0. The overfitting test of the model was carried out by using the permutation test method. The *p*-value was calculated by using a statistical test, and the OPLS-DA dimensionality reduction method was used to calculate variable importance in projection (VIP). Fold change was used to calculate the difference multiple between groups, all to measure the influence strength and interpretative ability of each metabolite’s content on sample classification and discrimination, as well as assisting with metabolite screening. Metabolites were considered statistically significant when the *p*-value was <0.05 and the VIP value was >1. Metabolite identification was carried out by using spectrogram databases, including HMDB, MassBank, LipidMaps, mzCloud, KEGG, and the Nomi Metabolism Standard Database. Metabolite pathway analysis was conducted by using MetobAnalyst 5.0 (https://www.Metaboanalyst.ca/MetaboAnalyst/ModuleView.xhtml, accessed on 23 March 2023) [27].

### 2.6. Statistical Analysis (Meat Quality)

The data normality was tested by using the Q-Q plot. The meat quality data were analyzed by using one-way ANOVA in SPSS version 26.0 (IBM). The model included fixed effects of treatments based on the following equation:

Y_ijk_ = μ + A_i_ +B_j_ + (AB)_ij_ + ε_ijk_;

where Y_ij_ is the dependent variable, µ is the overall mean, A_i_ is the fixed effect of the dietary energy levels, B_j_ is the fixed effect of the protein levels, (AB)_ij_ is the interaction of the energy and protein levels, and ε_ijk_ is the residual error associated with each Y_ijk_. The means presented in Table 2 were separated by using Tukey’s test with significance set to *p* < 0.05.

## 3. Results

### 3.1. Meat Quality Characteristics

According to Table 2, the pH value was greater for goats fed the LELP diet across the treatments. The dietary energy levels significantly affected (*p* < 0.05) meat tenderness (shear force) and brightness (L*) across the treatments, regardless of the dietary protein levels. There was a significant (*p* < 0.05) interaction between dietary energy and protein levels for meat tenderness. The meat color in the LEHP-fed group was brighter (L*) than in the other three groups. The shear force study revealed that the HEHP-fed group had significantly more tender meat (*p* < 0.05) than the LEHP-fed group.

### 3.2. Analysis of Volatile Flavor Substances of Meat by GC-MS

**Figure 1:**
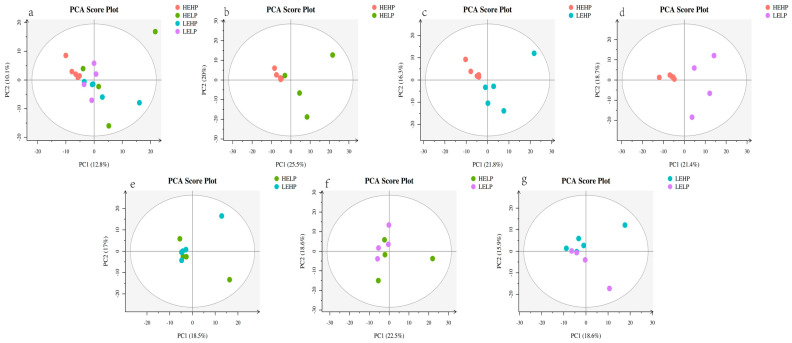
PCA score charts: score chart of HEHP, HELP, LEHP, and LELP groups (**a**), score chart of HEHP and HELP group (**b**), score chart of HEHP and LEHP group (**c**), score chart of HEHP and LELP group (**d**), score chart of HELP and LEHP group (**e**), score chart of HELP and LELP group (**f**), and score chart of LEHP and LELP group (**g**).

**Figure 2:**
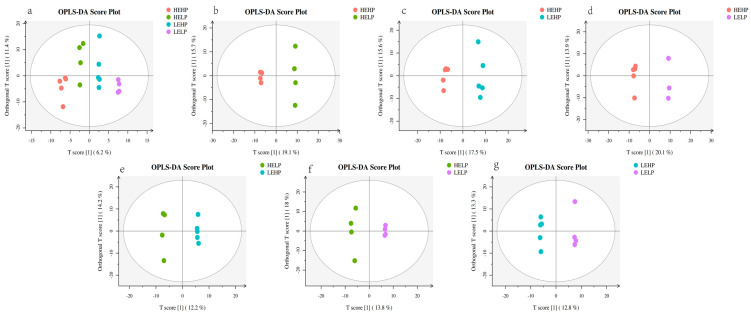
OPLS-DA score charts: score chart of HEHP, HELP, LEHP, and LELP groups (**a**), score chart of HEHP and HELP group (**b**), score chart of HEHP and LEHP group (**c**), score chart of HEHP and LELP group (**d**), score chart of HELP and LEHP group (**e**), score chart of HELP and LELP group (**f**), and score chart of LEHP and LELP group (**g**).

**Figure 3:**
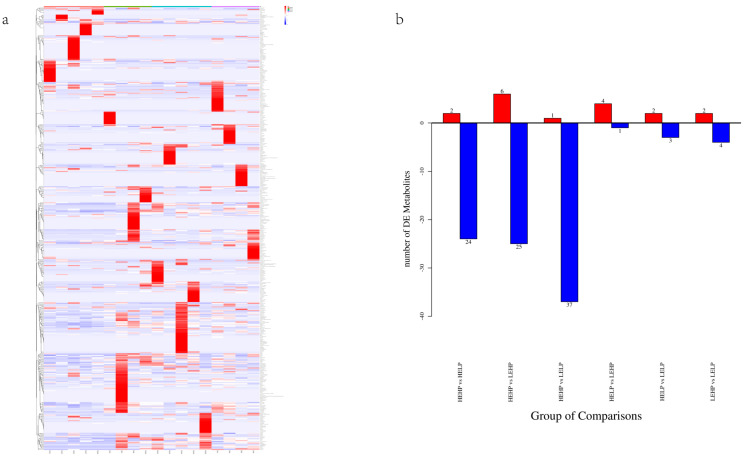
Heat map (**a**) and statistical analysis (**b**) of detected volatile compounds in the muscle of Yunshang black goats fed diets with different dietary energy and protein levels. The statistical analysis (**b**) shows red bars representing the number of differentiated metabolites between different groups that were up-regulated, and blue bars representing the number of downregulated metabolites among different groups.

The PCA analysis (Figure 1) revealed no significant differentiation among the four groups. Orthogonal partial least squares discriminant analysis (OPLS-DA) corroborated the dispersion of principal components among the four meat groups (Figure 2b–g), with PC1 accounting for 11.4% of the variation and PC2 accounting for 6.2% (Figure 2a). The heat map detected 493 volatile substances and demonstrated the varied metabolite composition of the four groups of mutton (Figure 3a). Among the four groups, 111 metabolites were significantly different (*p* < 0.05) (Figure 3b). For volatile organic compounds (VOCs), the levels of 2 compounds were higher in the HEHP group compared with the HELP group (*p* < 0.05), whereas those of 24 compounds were lower. Compared with the LEHP group, the HEHP group had 6 compounds at a higher level and 25 at a lower level (*p* < 0.05). The level of 1 compound in the HEHP group was higher than in the LELP group, while those of 37 compounds were lower (*p* < 0.05). In comparison with the LEHP group, the HELP group had four compounds at a higher level and one at a lower level (*p* < 0.05). In comparison with the LELP group, the HELP group had two compounds at a higher level and three at a lower level (*p* < 0.05). The levels of two chemicals were greater in the LEHP group compared with the LELP group, whereas those of four compounds were lower (*p* < 0.05). A total of 72 flavor compounds with CAS annotations were identified (Table 3), including alkanes (20), alcohols (13), ketones (12), acids (7), aromatic compounds (6), aldehydes (4), esters (3), ethers (3), phenols (2), and N-containing compounds (2).

### 3.3. Analysis of Flavor Substances of Meat by LC-MS

**Figure 4:**
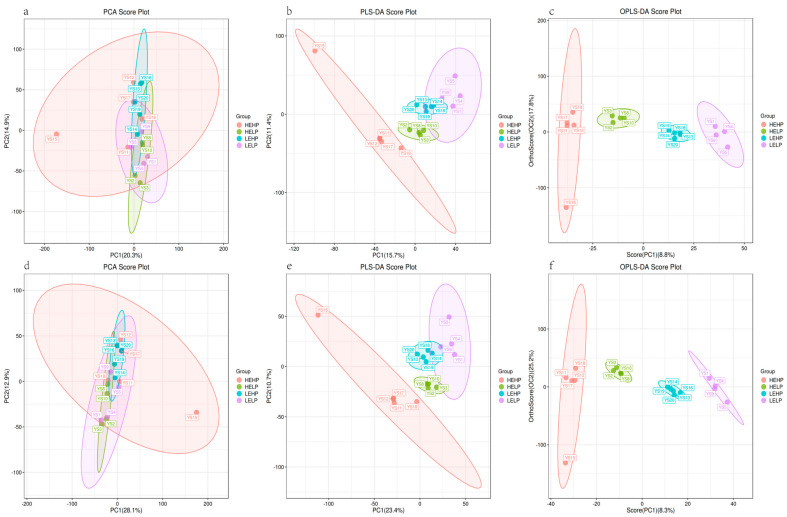
Metabolomics analysis of meat samples of the Yunshang black goats fed diets with different dietary energy and protein levels. Principal component analysis (PCA) scoring plots based on positive ion modes (**a**) and negative ion modes (**d**), partial least squares discriminant analysis (PLS-DA) scoring plots based on positive ion modes (**b**) and negative ion modes (**e**), and orthogonal partial least squares discriminant analysis (OPLS-DA) scoring plots based on positive ion modes (**c**) and negative ion modes (**f**).

**Figure 5:**
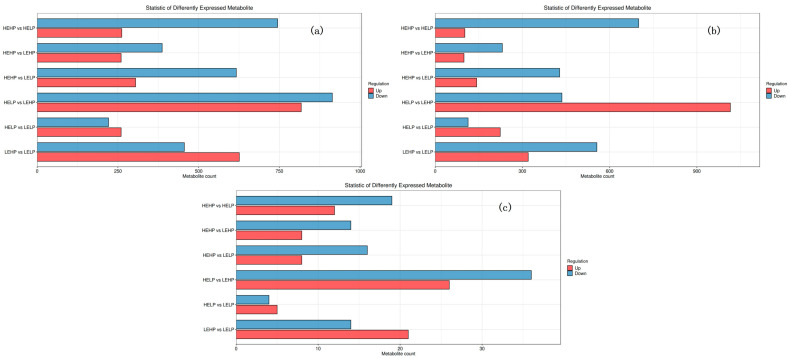
Columnar statistical diagram of differential metabolites of meat samples of the Yunshang black goats fed diets with different dietary energy and protein levels. Differential metabolite column diagram in positive ion mode (**a**), differential metabolite column diagram in negative ion mode (**b**), and secondary differential metabolite screening column (**c**).

**Figure 6:**
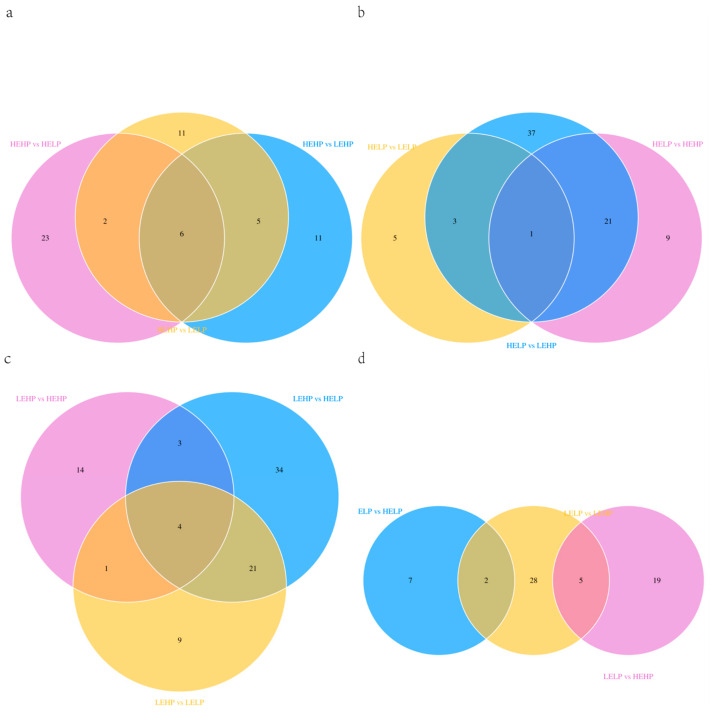
Venn diagram that illustrates the overlap of differential metabolites (DFMs) related to the four groups based on LC-MS analysis of raw mutton from goats fed diets with different dietary energy and protein levels (e.g., HEHP-related DFMs refer to the mutual DFMs between HEHP and the other three groups). DFMs associated with HEHP (**a**); DFMs related to HELP (**b**); DFMs related to LEHP (**c**); DFMs associated with LELP (**d**).

LC-MS detection in both positive and negative ion modes was used to understand the changes in the metabolomic profile of meat from goats fed diets with varied energy and protein levels. The samples from four groups were analyzed by using multivariate statistical approaches such as PCA, PLS-DA, and OPLS-DA (Figure 4). PCA analysis of the results in positive ion mode (Figure 2a) and negative ion mode (Figure 2d) revealed that the four sample groups overlapped, with insufficient separation. We then employed PLS-DA and OPLS-DA, which could perfectly identify all samples based on the positive ion pattern (Figure 2b,c) and the negative ion pattern (Figure 2e,f). Corona discharge is more likely to occur under electrospray conditions in negative ion mode than in positive ion mode. Variables such as pKa and surface activity influence the ionizability of ions in positive ion mode but have different effects in negative ion mode [28]. The positive ion mode had more differential metabolites than the negative ion mode, with 6712 and 4976 differential metabolites in each of the six comparison groups, respectively (Figure 5a,b). Secondary differential metabolites were selected for 348 metabolites (Figure 5c). Table 4 shows that the HEHP group had more DFMs (VIP > 1, *p* < 0.05) than the HELP group, with 12 up-regulated and 19 down-regulated DFMs. There were 22 DFMs in the HEHP and LEHP groups, with 8 being up-regulated and 14 down-regulated in the HEHP group. There were 24 DFMs in the HEHP and LELP groups, with 8 up-regulated and 16 down-regulated in the HEHP group. A total of 62 DFMs were found in the HELP and LEHP groups, with 26 being up-regulated and 36 down-regulated. A total of nine DFMs were found in the HELP and LELP groups, with five being up-regulated and four down-regulated. There were 35 DFMs in the LEHP group compared with the LELP group, with 21 being up-regulated and 14 down-regulated. The Venn diagram showed six distinct metabolites related to the HEHP group: L (-)-carnitine, l-glutamine, L-kynurenine, anabasine, saccharopine, and 9(S)-HPODE (Figure 4a). One distinct metabolite, L-tyrosine, was linked to the HELP group (Figure 4b). The LEHP group was connected with four distinct metabolites: L-leucine, acetylphosphate, cerulenin, and 9(S)-HPODE (Figure 6c). There were no differential metabolites linked with the LELP group (Figure 6d).

## 4. Discussion

It is well established that several factors associated with animals (breed, species, genetic makeup, and health) and diet (feeding sources, ingredient included, and their varying chemical characteristics, particularly energy and protein contents) affect fattening performance and meat quality [29]. Meat pH, drip loss, cooking loss, shear force, and color are important biomarkers used to evaluate meat quality and consumer preference [30]. Nowadays, using accurate and more detailed techniques such as GC-MS can provide greater insights into how different food compositional factors can affect meat quality and, in particular, the metabolomic profile [31]. As a result, this experiment could serve as a baseline for understanding how variations in dietary energy and protein contents affect meat quality and the meat metabolome. In this experiment, the meat pH was significantly different between the LEHP and LELP groups, suggesting that dietary energy and protein contents influence meat quality parameters. However, the recorded pH in this experiment was in the normal range (between 6.14 and 6.54), indicating good-quality meat production [32]. Changes in meat pH can be attributed to dynamic changes in lactic acid production, as several studies have documented that dietary factors directly influence lactic acid production and its muscular accumulation rate. Our findings are in line with Peng et al. [33], who reported that feeding higher protein levels resulted in a decrease in meat pH in pigeons. Parameters like cooking and drip losses determine the water-holding capacity of meat. In this experiment, there were no significant variations in drip and cooking losses among groups, indicating that the meat had higher water-holding capacity, which allows for a longer shelf life and a better protein structural arrangement. These findings are consistent with prior research, which found that dietary energy and protein intake did not affect the water-holding capacity of sheep, goat, and bovine meat. However, a study by Su et al. [34] reported a decrease in cooking and drip losses in the meat of fattening Tibetan sheep fed different dietary energy and protein levels. It is widely recognized that feeding an optimal dietary energy and protein ratio results in greater muscle glycogen buildup and marbling, which improves water-holding capacity. Thus, we may infer from this experiment that dietary energy and protein levels increase the bioenergetics of fattening Yunshang black goats.

Tenderness is a desirable meat attribute that not only determines meat price but also consumer preferences by making meat softer, easier to chew, and tastier [35]. The tenderness of meat can be affected by various characteristics, including muscle mass, connective tissue content, marbling, and processing procedures. Typically, shear force can directly reflect the tenderness of the meat; the smaller the shear force, the better the tenderness [36]. This study found significant differences in shear force among groups when dietary protein and energy levels were changed. These results are consistent with the study conducted by Erasmus et al. [37], who reported that Duper lambs fed diets with different chemical compositions showed significant changes in meat tenderness. However, studies by Lingyan Li et al. [38] revealed that different dietary energy and protein levels did not affect the shear force in F1 Angus × Chinese Xiangxi yellow cattle. It is speculated that the difference may be caused by the different energy and protein levels changing the type and proportion of newly synthesized collagen in skeletal muscle, reducing muscle maturity and thus negatively regulating shear force [39,40]. Meat color is an important reference index for consumers when buying meat. Increasing redness in the meat suggests muscle enrichment with hemoglobin and myoglobin. Dietary nutrition is a critical factor influencing the quality of raw meat, particularly its fatty acid composition [41]. Small water-soluble metabolites, such as free amino acids, nucleotides, peptides, sugars, thiamine, and fatty acids, play a crucial role as meat flavor precursors in reactions like the Maillard and Strecker reactions [42]. To better understand how different dietary protein and energy levels improve meat quality, this study utilized non-targeted metabolomics methods using LC-MS and GC-MS. We identified several metabolites that could serve as potential biomarkers to distinguish different feeding methods and indicate meat quality. Our LC-MS analysis revealed that L-carnitine and its esters help reduce oxidative stress and have been proposed for treating various conditions, such as heart failure, angina, and weight loss [43].

Studies have found that providing L-carnitine during the fattening period of lambs with early feed restriction can alter meat quality traits by increasing lightness, oxidative stability, and intramuscular fat content, although it can worsen the fatty acid profile [44]. L-glutamine (Gln), a non-essential amino acid with an umami or sweet taste, can decompose into glutamine or propyl esterification to pyrrole carboxyl alcohol in alcohol, alkali, or hot water [22]. Adding Gln to the diet can mitigate the negative effects of heat stress on growth performance, carcass characteristics, meat quality, and meat color stability in broilers [45]. Anabasine, a trace alkaloid in tobacco products, can be teratogenic at high doses in animals [46]. L-kynurenine, a metabolite produced during tryptophan metabolism, can increase reactive oxygen species (ROS) in muscle cells, leading to muscle atrophy [47,48]. Saccharopine, an intermediate product in the lysine decomposition pathway, can cause developmental delays and death in mice if lysine metabolism is abnormal [49]. Compared with the other three diet groups, the HEHP group showed increased levels of L-carnitine and anabasine but decreased levels of L-glutamine, saccharopine, and L-kynurenine, indicating a decline in meat quality. L-tyrosine and L-leucine, associated with sweetness, contribute to flavor through thermal reactions with sugar, producing fruit and fatty aromas, respectively [50]. Acetyl phosphate, which produces acetic acid in fermented sausages, can result in poor flavor [51]. Cerulenin is a natural antibiotic that inhibits fatty acid synthesis [52]. In this experiment, the HELP group had higher L-tyrosine levels than the HEHP group but lower than the LEHP and LELP groups, with the LEHP group having the highest L-tyrosine content. The LEHP group also had the highest levels of L-leucine and acetyl phosphate but lower cyanobacterin content compared with the other groups. Metabolomics analyses identified L-glutamine, L-tyrosine, and L-leucine as potential markers for improving meat flavor in Yunshang black goats and distinguishing different dietary protein and energy feeding levels. The fatty part of meat, especially the phospholipid part, undergoes autoxidation and produces a large number of volatiles, such as fatty acids, aldehydes, ketones, alcohols, nitrogen, and sulfur compounds, which are odor-active compounds and have a great impact on the flavor of cooked meat [53]. Based on GC-MS analysis, 72 volatile compounds were found in this experiment, including alkanes (20), alcohols (13), ketones (12), fatty acids (7), aromatic compounds (6), aldehydes (4), esters (3), ethers (3), phenols (2), and N-containing compounds (2). It was found that there were more differential metabolites in the HEHP group, HELP group, LEHP group, and LELP group and that the content of most volatile compounds was higher than in the HEHP group. Studies have shown that methyl mercaptan is a common sulfur-containing spice and has a strong influence on the flavor of steamed silver carp [54]. The content of differential flavor compounds, including methyl mercaptan, 2,4-dimethyl-heptane, and acetone in the HELP, LEHP, and LELP groups, was very high, with significant differences. The results suggest that diets with different energy and protein contents may alter meat flavor by producing different volatile compounds. Few studies have found that with the increase in dietary protein supply from low to high, the dry matter content of *longispectoral* muscle increases, while the heme and lipid contents decrease [55]. In addition, the growth performance, meat yield, and meat quality of fattened yaks can be improved by increasing dietary energy concentration [56]. However, there has been little research on the correlation between different dietary protein and energy contents and the quality and flavor of meat. In this experiment, four groups of goats were fed diets with different dietary energy and protein levels, and it was found that these levels influenced their volatile compounds and hydrophilic metabolites. The results show that feeding diets with different protein and energy levels had a great influence on meat flavor. However, the important role and function of these metabolites in meat flavor need to be further studied.

## 5. Conclusions

Based on the results, it can be concluded that dietary energy and protein levels significantly impact goat meat quality, particularly in terms of flavor and fatty acid content. In this study, feeding a high-energy and protein-containing diet improved the tenderness of the meat. According to metabolomics analyses (LC-MS and GC-MS), the metabolomic profile of goat meat changed with different protein and energy levels in the diet. The hydrophilic components and volatile substances in raw mutton are significantly influenced by dietary energy and protein levels. Overall, we can conclude that feeding strategies based on different dietary energy and protein levels can alter goat meat quality and its flavor. Further investigation regarding specific dietary energy and protein sources is needed to help improve meat quality.

## Figures and Tables

**Table 1:** Diet formulation and chemical composition on a dry basis.

Ingredient List	Diets ^1^			
	HEHP	HELP	LEHP	LELP
Ground corn grains (%) Wheat bran (%)	33.00 1.40	38.45 0.00	29.50 5.40	31.00 7.40
Soybean meal (%)	12.65	9.20	12.10	9.40
Corn silage (%)	43.00	41.00	0.00	0.00
Whole triticale plant (%)	0.00	0.00	14.00	10.00
Wheat straw (%)	7.00	9.00	36.00	40.00
Calcium carbonate (%)	0.60	0.60	0.70	0.70
NaCl (%)	0.50	0.50	0.50	0.50
Sodium bicarbonate (%)	0.25	0.25	0.10	0.00
Mineral and vitamin Premix ^2^ (%) Feed graded urea (%)	1.00 0.60	1.00 0.00	1.00 0.70	1.00 0.00
Forage to concentrate ratio	50:50	50:50	50:50	50:50
Chemical Composition				
Dry matter (%)	64.74	67.01	74.68	73.75
Metabolizable energy ^3^ (MJ/kg)	9.74	9.76	8.18	8.14
Crude protein (%)	12.99	10.01	13.04	10.05
NDF ^4^ (%)	30.43	30.82	45.82	47.08
ADF ^5^ (%)	15.52	15.72	24.80	25.36
Calcium (%)	0.59	0.58	0.59	0.60
Phosphorous (%)	0.33	0.31	0.29	0.29

^1^ Diets were (1) high energy with high protein (HEHP), (2) high energy with low protein (HELP), (3) low energy with high protein (LEHP), and (4) low energy with low protein (LELP). ^2^ Each kg of mineral and vitamin premix contained: VA, 10,000 IU; VD, 1000 IU; VE, 200 IU; Fe, 145 mg; Zn, 80 mg; Cu, 20 mg; Mn, 98 mg; I, 2.5 mg; Se, 0.35 mg; and Co, 0.65 mg. ^3^ The metabolizable energy of the diets was calculated according to the China Feeding Standard of Meat Producing Sheep and Goat (NY/T816—2021) [17]. ^4^ NDF: neutral detergent fiber. ^5^ ADF: acid detergent fiber.

**Table 2:** Effects of different dietary energy and protein levels on the meat quality parameters of the Yunshang black goat.

Variables	Diets ^1^					*p*-Value ^2^		
	HEHP	HELP	LEHP	LELP	SEM	E	P	E*P
pH	6.232 ^ab^	6.284 ^ab^	6.14 ^b^	6.54 ^a^	0.0591	0.449	0.046	0.117
Drip loss	59.93 ^ab^	60.49 ^ab^	58.95 ^b^	61.10 ^a^	0.3488	0.779	0.041	0.214
Cooking loss	40.07 ^ab^	39.51 ^ab^	41.05 ^a^	38.90 ^b^	0.3478	0.779	0.053	0.241
Shear force	86.16 ^b^	95.41 ^a^	91.98 ^a^	82.16 ^b^	1.4080	0.044	0.869	<0.001
Brightness (L*)	21.37 ^b^	22.02 ^ab^	23.03 ^a^	22.09 ^ab^	0.2355	0.051	0.735	0.076
Redness (a*)	11.76	11.18	11.99	11.34	0.2121	0.658	0.172	0.937
Yellowness (b*)	2.17	2.39	2.59	2.19	0.0876	0.534	0.619	0.092

^1^ Diets were (1) high energy with high protein (HEHP), (2) high energy with low protein (HELP), (3) low energy with high protein (LEHP), and (4) low energy with low protein (LELP). ^2^ *p*-Value represents E = energy levels, P = protein levels, and E*P = interaction of energy and protein levels. Note: The lowercase letters in the shoulder labels of peer data indicate significant differences (*p* < 0.05), while the same letters or no letters in the shoulder labels indicate no significant differences (*p* > 0.05).

**Table 3:** GC-MS analysis of volatile components in raw meat of Yunshang black goats fed diets with different dietary energy and protein levels.

Metabolite	CAS	RT	VIP	Log2FC	*p*-Value
HEHP vs. HELP					
Tridecane, 3-methyl-	6418-41-3	16.251	1.945	−2.201	0.0037
Methylcyclopentane	96-37-7	1.883	1.862	−4.157	0.0076
4-Xylene	106-42-3	8.829	1.839	−2.418	0.0091
n-Dodecane	112-40-3	11.167	1.819	−2.395	0.0105
n-Decane	124-18-5	5.013	1.818	−2.151	0.0106
4,6-Dimethyldodecane	61,141-72-8	12.272	1.810	−3.134	0.0112
Methyl mercaptan	74-93-1	1.876	1.807	−29.092	0.0114
Undecane, 3-methyl-	1002-43-3	10.016	1.784	−1.986	0.0133
n-Tetradecane	629-59-4	17.157	1.771	−2.079	0.0144
2,4-Di-tert-butylphenol	96-76-4	36.466	1.755	−2.422	0.0159
Trimethylamine	75-50-3	1.788	1.722	−29.020	0.0194
Heptane, 2,4-dimethyl-	2213-23-2	2.356	1.693	−2.039	0.0227
2-Phenoxyethanol	122-99-6	33.359	1.687	−1.858	0.0234
2-Octanone	111-13-7	13.705	1.673	−5.639	0.0253
5-Acetyldihydrofuran-2(3h)-one	29,393-32-6	31.745	1.669	−1.639	0.0258
Acetic acid	64-19-7	18.355	1.637	−2.936	0.0303
3-Hexanone	589-38-8	6.344	1.607	−3.518	0.0350
2-Methylundecane	7045-71-8	9.724	1.605	−3.452	0.0353
Acetone	67-64-1	2.379	1.604	−2.350	0.0355
3-Ethylpentane	617-78-7	2.128	1.601	−24.976	0.0359
Octanoic acid	124-07-2	31.999	1.600	1.017	0.0361
n-Hexadecane	544-76-3	13.358	1.591	−3.685	0.0376
Benzene, 1,3-bis(1,1-dimethylethyl)-	1014-60-4	17.701	1.581	−1.180	0.0394
Methyl heptyl ketone	821-55-6	16.705	1.552	−10.453	0.0447
Pentanal	110-62-3	4.369	1.552	22.173	0.0447
2,5-Dimethylnonane	17,302-27-1	5.201	1.528	−24.119	0.0495
HEHP vs. LEHP					
Nonanoic acid	112-05-0	34.009	2.283	1.398	0.000
1-Nonanol	143-08-8	23.644	2.115	−2.273	0.001
1-Phenyl-2-butanone	1007-32-5	26.552	2.062	−1.719	0.002
2,4-Di-tert-butylphenol	96-76-4	36.466	2.038	−1.784	0.002
2-Heptanol	543-49-7	14.999	1.991	−22.148	0.003
4-Sec-butyl-2,6-di-tert-butylphenol	17,540-75-9	29.248	1.967	−3.588	0.004
Naphthalene	91-20-3	25.037	1.952	17.123	0.004
Methyl mercaptan	74-93-1	1.876	1.952	−28.844	0.004
Heptane, 2,4-dimethyl-	2213-23-2	2.356	1.907	−1.439	0.006
Trimethylamine	75-50-3	1.788	1.904	−29.428	0.006
Sarcosine	107-97-1	1.663	1.903	−28.046	0.006
1,4-Butanediol	110-63-4	29.265	1.860	−2.807	0.009
Isobutyl alcohol	78-83-1	8.064	1.826	−7.004	0.011
2-Tridecanone	593-08-8	26.896	1.825	−18.699	0.011
n-Tetradecane	629-59-4	17.157	1.791	−2.499	0.014
Acetone	67-64-1	2.379	1.784	−1.384	0.014
Dodecanoic acid	143-07-7	39.527	1.781	−1.432	0.014
Methylthioethanol	5271-38-5	20.287	1.777	−22.802	0.015
Octylaldehyde	124-13-0	13.780	1.749	22.566	0.017
Palmitic acid	1957-10-3	44.605	1.739	−1.705	0.018
Pentylcyclopentane	3741-00-2	7.058	1.731	−2.185	0.019
2-Pentanone	107-87-9	4.351	1.692	−4.081	0.023
Pentanal	110-62-3	4.369	1.684	22.173	0.024
Acetic acid	64-19-7	18.355	1.675	−3.082	0.025
Formamide	1975-12-7	26.237	1.668	−2.730	0.026
Nonanal	124-19-6	16.797	1.604	5.258	0.035
Benzene, 1,3-bis(1,1-dimethylethyl)-	1014-60-4	17.701	1.591	−1.369	0.037
3-Octanol	589-98-0	17.040	1.575	−3.802	0.040
Isobornyl acrylate	5888-33-5	24.530	1.538	−1.799	0.047
1-Methoxy-2-hydroxypropane	107-98-2	9.001	1.531	19.428	0.048
n-Decane	124-18-5	5.013	1.530	−1.562	0.048
HEHP vs. LELP					
Nonanoic acid	112-05-0	34.009	2.124	1.648	0.000
Undecane, 3-methyl-	1002-43-3	10.016	2.118	−1.303	0.000
Ethylbenzene	100-41-4	8.365	2.066	−1.306	0.000
(4s)-4,6-Dimethyl-2-heptanone	790,248-21-4	12.455	2.053	−19.619	0.000
n-Dodecane	112-40-3	11.167	2.047	−1.502	0.001
n-Tetradecane	629-59-4	17.157	2.024	−2.007	0.001
2-Bromododecane	13,187-99-0	12.519	1.945	−2.151	0.002
4,6-Dimethyldodecane	61,141-72-8	12.272	1.941	−2.735	0.002
Heptane, 2,4-dimethyl-	2213-23-2	2.356	1.940	−1.484	0.002
Acetone	67-64-1	2.379	1.934	−1.687	0.003
Methylthioethanol	5271-38-5	20.287	1.909	−21.756	0.003
2-Octanone	111-13-7	13.705	1.906	−4.940	0.003
Methanol	67-56-1	3.116	1.883	−0.576	0.004
1,4-Butanediol	110-63-4	29.265	1.883	−2.686	0.004
Palmitic acid	1957-10-3	44.605	1.796	−1.747	0.009
Methyl ethyl ketone	78-93-3	3.103	1.768	−1.381	0.011
Methyl mercaptan	74-93-1	1.876	1.764	−27.945	0.011
2,5-Dimethylnonane	17,302-27-1	5.201	1.720	−23.112	0.015
Tridecane, 3-methyl-	6418-41-3	16.251	1.692	−1.835	0.018
Phenol	108-95-2	30.689	1.670	−1.580	0.020
Octane, 2,3,6,7-tetramethyl-	52,670-34-5	5.883	1.646	−1.866	0.023
2-Heptanol	543-49-7	14.999	1.641	−21.430	0.024
2-Phenoxyethanol	122-99-6	33.359	1.631	−2.263	0.025
n-Decane	124-18-5	5.013	1.621	−1.131	0.027
Formamide	1975-12-7	26.237	1.608	−3.196	0.029
Isovaleric acid	503-74-2	23.761	1.607	−7.175	0.029
Dimethyl trisulfide	3658-80-8	16.113	1.605	−19.885	0.029
Isobornyl acrylate	5888-33-5	24.530	1.597	−2.278	0.030
1,3,5-Trimethylbenzene	108-67-8	13.325	1.566	−3.454	0.035
Indole	120-72-9	38.444	1.566	−23.654	0.035
2-Pentanol	6032-29-7	8.954	1.558	−3.153	0.037
Sarcosine	107-97-1	1.663	1.558	−27.671	0.037
2-Methylheptane	592-27-8	2.110	1.552	−2.382	0.038
4-Ethylcyclohexanone	5441-51-0	15.193	1.518	−20.005	0.044
3-Ethylpentane	617-78-7	2.128	1.495	−23.998	0.048
4-Methyloctane	2216-34-4	2.713	1.495	−0.902	0.049
Isoamyl alcohol	123-51-3	11.596	1.493	−27.731	0.049
2-Nonanol	628-99-9	20.311	1.490	−21.356	0.050
HELP vs. LEHP					
Octyl formate	112-32-3	21.219	2.114	19.818	0.023
1-Octanol	111-87-5	21.218	2.059	−20.238	0.029
Methylcyclopentane	96-37-7	1.883	2.033	2.219	0.032
Benzyl alcohol	100-51-6	28.143	1.983	2.021	0.039
1,1,2-Trichloroethane	79-00-5	12.854	1.944	24.178	0.045
HELP vs. LELP					
Ethylbenzene	100-41-4	8.365	2.067	−1.041	0.028
5-Acetyldihydrofuran-2(3h)-one	29,393-32-6	31.745	2.048	2.134	0.031
(4s)-4,6-Dimethyl-2-heptanone	790,248-21-4	12.455	2.040	−2.226	0.032
Isophorone	78-59-1	21.876	1.986	−3.463	0.039
Methylcyclopentane	96-37-7	1.883	1.940	2.247	0.046
LEHP vs. LELP					
(4s)-4,6-Dimethyl-2-heptanone	790,248-21-4	12.455	2.572	−19.619	0.000
Butyrolactone	96-48-0	22.495	2.274	2.172	0.008
1,1,2-Trichloroethane	79-00-5	12.854	2.106	−24.138	0.019
1-Nonanol	143-08-8	23.644	2.070	1.130	0.023
Ethylbenzene	100-41-4	8.365	1.989	−1.110	0.032
4-Ethylcyclohexanone	5441-51-0	15.193	1.901	−20.005	0.044

**Table 4:** LC-MS analysis of volatile components of raw meat from Yunshang black goats fed diets with different dietary energy and protein levels.

Metabolites	*m*/*z*	RT	VIP	Log2FC	*p*-Value	P/N
HEHP vs. HELP						
1-Palmitoylglycerophosphocholine	496.33	516.70	1.78	−0.92	0.00788	P
L-Kynurenine	209.09	102.60	1.83	0.90	0.00903	P
Butyryl-L-carnitine	232.15	148.20	2.02	−3.94	0.00965	P
L-Glutamine	145.06	70.30	1.65	−1.23	0.01027	N
L-Methionine	150.06	63.10	1.66	−1.27	0.01065	P
3′-AMP	346.06	49.70	1.81	−4.11	0.01113	N
N2-gamma-Glutamylglutamine	274.10	46.90	1.94	−4.02	0.01344	N
Oleic acid	283.26	558.60	1.73	−1.63	0.01414	P
Ethylmethylacetic acid	102.13	684.90	1.86	−0.55	0.01500	P
9(S)-HPODE	313.24	413.60	1.92	−1.83	0.01652	P
Aminosalicylic acid	152.04	381.30	1.82	−1.81	0.01921	N
16(R)-HETE	319.23	504.30	1.37	−0.46	0.02097	N
Cholesterol sulfate	466.31	573.10	1.43	−1.20	0.02345	N
L-Tyrosine	182.08	65.40	1.86	2.00	0.02740	P
Anabasine	144.98	687.30	1.74	−0.79	0.02857	P
Saccharopine	276.11	58.70	1.68	1.22	0.03205	P
Phenylacetylglycine	194.08	273.50	1.52	−0.74	0.03426	P
Palmitoylethanolamide	300.29	587.30	1.70	1.22	0.03440	P
Uridine	245.08	62.80	1.83	4.30	0.03815	P
N-Alpha-acetyllysine	188.07	190.40	1.71	−1.06	0.03882	P
2-Pyrrolidinone	100.08	102.90	1.80	0.80	0.04024	P
L(-)-Carnitine	162.11	161.30	1.63	0.40	0.04103	P
3-Methyladenine	149.02	408.80	1.45	−0.77	0.04162	P
S-Inosyl-L-homocysteine	386.11	58.80	1.71	2.24	0.04309	P
Prostaglandin I2	353.23	344.50	1.70	−1.30	0.04342	P
Palmitoyl-L-carnitine	400.34	653.10	1.68	−0.76	0.04610	P
10-Hydroxydecanoic acid	187.13	334.10	1.64	2.27	0.04618	N
2-Aminophenol	110.02	85.50	1.77	0.83	0.04631	P
Nalpha-Methylhistidine	152.08	45.00	1.76	0.82	0.04635	P
(S)-2-Methylmalate	148.04	56.60	1.75	0.99	0.04713	P
D-Ribose	151.04	43.00	1.62	−0.63	0.04965	P
HEHP vs. LEHP						
Stearic acid	284.18	252.40	2.59	−4.48	0.00002	P
Acetylphosphate	139.98	88.30	2.44	−1.98	0.00040	P
L-Sorbose	179.99	638.40	2.52	−2.50	0.00076	N
L(-)-Carnitine	162.11	161.30	2.34	0.76	0.00160	P
Saccharopine L-Kynurenine	276.11 209.09	58.70	2.21	2.40	0.00410	P
		102.60	2.23	1.20	0.00434	P
Hypoxanthine	137.05	64.90	2.16	−1.92	0.00623	P
Sodium deoxycholate	414.32	566.00	2.07	−1.89	0.01191	P
Hippuric acid	178.05	104.70	2.35	−1.86	0.01202	N
L-Glutamine	145.06	70.30	2.01	−0.84	0.02246	N
Allocystathionine	222.08	230.50	1.90	0.57	0.02296	P
Equol	241.08	92.40	2.03	−1.05	0.02297	N
Cytidine	244.09	53.90	1.90	0.56	0.02334	P
Guanidinosuccinic acid	174.96	89.10	1.97	−1.79	0.02848	N
Anabasine	144.98	687.30	1.86	−0.68	0.03134	P
Cerulenin	224.12	472.10	1.78	1.73	0.04027	P
Succinic acid semialdehyde	103.04	51.10	1.78	1.08	0.04224	P
L-Leucine	132.10	65.70	1.77	−0.79	0.04292	P
Dodecanedioic acid	229.14	269.30	1.85	−1.17	0.04597	N
Mannitol	182.99	32.70	1.74	0.22	0.04629	P
Epsilon-caprolactam	114.09	208.30	1.74	−1.98	0.04751	P
9(S)-HPODE	313.24	413.60	1.91	−1.18	0.04999	P
HEHP vs. LELP						
Daidzin	397.22	473.40	2.11	−2.53	0.00214	N
Saccharopine	276.11	58.70	2.06	1.80	0.00327	P
L(-)-Carnitine	162.11	161.30	2.06	0.74	0.00340	P
Erythritol	121.03	271.00	2.10	1.78	0.00674	N
Sodium deoxycholate	414.32	566.00	1.99	−2.41	0.00739	P
Hypoxanthine	137.05	64.90	1.96	−1.65	0.00888	P
Glycitein	284.07	383.70	1.99	−2.46	0.00908	N
Succinic acid	117.02	45.80	1.97	1.51	0.00911	N
L-Kynurenine	209.09	102.60	1.92	0.68	0.01052	P
AMP	348.07	209.70	1.85	1.58	0.01247	P
Oleic acid	283.26	558.60	1.88	−1.56	0.01345	P
3-Methyladenine	149.02	408.80	1.87	−1.01	0.01418	P
9(S)-HPODE	313.24	413.60	1.86	−1.76	0.01479	P
L-Glutamine	145.06	70.30	1.85	−1.75	0.01554	N
Mannitol	182.99	32.70	1.87	0.28	0.01727	P
Allocholic acid	408.37	633.40	1.76	−1.40	0.02448	P
Dodecanedioic acid	229.14	269.30	1.74	−0.98	0.03112	N
Dimethirimol	209.15	452.50	1.70	−1.65	0.03312	P
Ecgonine methyl ester	200.13	65.40	1.74	−0.67	0.03421	P
L-Sorbose	179.99	638.40	1.75	−1.74	0.03576	N
(6Z)-Octadecenoic acid	282.28	617.40	1.68	−1.71	0.04401	P
Glutathione	307.08	56.40	1.62	−4.08	0.04496	P
Anabasine	144.98	687.30	1.64	−0.92	0.04895	P
HELP vs. LEHP						
10-Hydroxydecanoic acid	187.13	334.10	2.00	−3.02	0.00001	N
L-Leucine	132.10	65.70	1.88	−1.21	0.00072	P
9(S)-HPODE	313.24	413.60	1.90	0.65	0.00118	P
Epiandrosterone	271.23	519.90	1.79	1.12	0.00229	N
3-Methyladenine	149.02	408.80	1.85	0.82	0.00243	P
L-Tyrosine	182.08	65.40	1.89	−2.82	0.00258	P
3-Ketosphingosine	280.26	501.40	1.80	−3.05	0.00295	P
Phloroglucinol	127.04	75.40	1.80	−3.09	0.00300	P
Arachidic acid	311.30	672.40	1.73	−1.64	0.00318	N
all-trans-Retinoic acid	299.26	545.50	1.84	1.29	0.00345	N
Quinolin-2-ol	146.06	265.40	1.75	−1.54	0.00421	P
Acetylphosphate	139.98	88.30	1.72	−1.50	0.00593	P
Nalpha-Methylhistidine	152.08	45.00	1.74	−0.97	0.00631	P
Cholesterol sulfate	466.31	573.10	1.69	1.58	0.00645	N
Aminosalicylic acid	152.04	381.30	1.70	2.34	0.00741	N
N2-gamma-Glutamylglutamine	274.10	46.90	1.69	4.43	0.00743	N
L-Methionine	150.06	63.10	1.75	1.31	0.00773	P
Uridine	245.08	62.80	1.69	−5.55	0.00831	P
Glycylleucine	189.12	133.70	1.68	−0.81	0.00902	P
2-Aminophenol	110.02	85.50	1.69	−0.96	0.00959	P
2-Pyrrolidinone	100.08	102.90	1.68	−0.97	0.00982	P
Cerulenin	224.12	472.10	1.70	2.19	0.01069	P
Phthalic acid	165.04	589.70	1.65	0.74	0.01084	N
5-Amino-2-oxopentanoic acid	131.05	65.10	1.68	−1.31	0.01091	P
1-Palmitoylglycerophosphocholine	496.33	516.70	1.64	0.97	0.01185	P
(S)-2-Methylmalate	148.04	56.60	1.66	−1.08	0.01202	P
L-Malic acid	133.01	44.40	1.69	−2.92	0.01328	N
gamma-Amino-gamma-cyanobutanoate	129.07	52.20	1.63	−0.94	0.01381	P
Myristic acid	227.20	478.70	1.58	−5.09	0.01385	N
D-Ribose	151.04	43.00	1.64	0.49	0.01420	P
L-Tryptophan	205.10	190.40	1.61	−1.24	0.01580	P
S-Inosyl-L-homocysteine	386.11	58.80	1.64	−2.30	0.01670	P
5′-Methylthioadenosine	298.10	160.10	1.55	−4.64	0.01748	P
Octadecanamide	284.29	667.50	1.60	0.92	0.01774	P
Tridemorph	298.31	659.80	1.57	−0.73	0.01803	P
Palmitoylethanolamide	300.29	587.30	1.58	−1.33	0.01847	P
Taurine	126.02	53.50	1.58	−1.25	0.02000	P
UMP	323.03	46.70	1.54	2.42	0.02036	N
Anhydrotetracycline	427.12	47.30	1.56	−5.07	0.02124	P
8-HETE	303.23	530.20	1.58	1.11	0.02173	P
Butyryl-L-carnitine	232.15	148.20	1.54	3.87	0.02306	P
Oleic acid	283.26	558.60	1.55	1.14	0.02467	P
Hydroquinone	110.02	348.20	1.51	−0.68	0.02557	P
o-Toluate	135.05	289.70	1.48	1.25	0.02569	N
Kyotorphin	320.16	253.60	1.53	−1.20	0.02741	P
Epsilon-caprolactam	114.09	208.30	1.56	−2.48	0.02790	P
N-Acetyl-L-aspartic acid	174.04	45.20	1.51	−0.75	0.02851	N
Spermine	203.22	41.80	1.56	−1.26	0.02872	P
N-Formyl-L-methionine	176.97	158.20	1.46	−1.32	0.03044	P
Pyrrolidonecarboxylic acid	130.05	513.40	1.56	1.17	0.03114	P
26-Hydroxyecdysone	480.28	348.50	1.47	0.78	0.03436	P
ATP	507.12	589.70	1.43	1.19	0.03842	N
3,4-Dihydroxyphenylpropanoate	165.05	65.30	1.42	−2.16	0.03940	P
Hypoxanthine	137.05	64.90	1.46	−1.53	0.04089	P
Linoleic acid	280.26	581.80	1.45	−0.50	0.04232	P
3′-AMP	346.06	49.70	1.40	3.03	0.04276	N
L (-)-Carnitine	162.11	161.30	1.39	0.36	0.04297	P
Isocitric acid	191.02	439.80	1.38	0.76	0.04392	N
N-Alpha-acetyllysine	188.07	190.40	1.46	0.80	0.04473	P
Nitrendipine	361.14	308.30	1.44	−0.28	0.04568	P
Creatinine	113.06	98.50	1.38	0.27	0.04841	P
HELP vs. LELP						
L-Tyrosine	182.08	65.40	2.21	−1.03	0.00582	P
L-Arginine	175.12	46.40	2.13	2.32	0.01009	P
4-Nitrophenol	138.02	335.00	2.18	1.57	0.01447	N
all-trans-Retinoic acid	299.26	545.50	1.96	0.53	0.02960	N
Glycylleucine	189.12	133.70	1.92	−1.11	0.03570	P
Oxidized glutathione	611.15	42.70	1.87	0.81	0.04211	N
S-(Hydroxymethyl)glutathione	338.10	56.50	1.90	0.86	0.04435	P
Nitrendipine	361.14	308.30	1.85	−0.31	0.04691	P
Procollagen 5-hydroxy-L-lysine	197.81	471.20	1.83	−1.34	0.04742	N
LEHP vs. LELP						
10-Hydroxydecanoic acid	187.13	334.10	2.12	2.63	0.00018	N
5-Guanidino-3-methyl-2-oxopentanoate	187.11	55.70	2.19	2.50	0.00025	P
3-Methyladenine	149.02	408.80	2.17	−1.06	0.00038	P
Phloroglucinol	127.04	75.40	2.14	4.06	0.00068	P
3-Ketosphingosine	280.26	501.40	2.13	2.76	0.00076	P
7-Dehydrodesmosterol	365.32	508.90	2.02	0.49	0.00269	P
5′-Methylthioadenosine	298.10	160.10	1.98	6.52	0.00315	P
Phenyl acetate	134.89	615.90	1.89	−1.23	0.00476	N
5-Amino-2-oxopentanoic acid	131.05	65.10	1.94	1.03	0.00591	P
Nalpha-Methylhistidine	152.08	45.00	1.95	0.85	0.00606	P
4,4′-Sulfonyldiphenol	249.02	326.70	1.82	−0.95	0.00661	N
(S)-2-Methylmalate	148.04	56.60	1.93	0.95	0.00719	P
Acetylphosphate	139.98	88.30	1.90	1.83	0.00725	P
2-Aminophenol	110.02	85.50	1.90	0.86	0.00836	P
2-Pyrrolidinone	100.08	102.90	1.85	0.86	0.01168	P
N-Acetyl-L-aspartic acid	174.04	45.20	1.76	1.04	0.01324	N
L-Tyrosine	182.08	65.40	1.82	1.79	0.01362	P
Cerulenin	224.12	472.10	1.87	−1.96	0.01571	P
9(S)-HPODE	313.24	413.60	1.78	−0.58	0.01706	P
UMP	323.03	46.70	1.73	−2.83	0.01897	N
Oleic acid	283.26	558.60	1.73	−1.07	0.02238	P
Pyrrole-2-carboxylic acid	111.02	601.40	1.73	−0.49	0.02304	P
3-Dehydroshikimate	172.98	83.40	1.76	−0.85	0.02398	P
N-Acetylornithine	173.09	53.30	1.77	−0.24	0.02506	N
Palmitoylethanolamide	300.29	587.30	1.63	1.11	0.03177	P
Dulcin	181.10	254.50	1.66	−0.23	0.03410	P
Uridine	245.08	62.80	1.64	4.15	0.03496	P
L-Leucine	132.10	65.70	1.62	0.88	0.03945	P
all-trans-Retinoic acid	299.26	545.50	1.53	−0.76	0.04014	N
Hippuric acid	178.05	104.70	1.83	2.01	0.04031	N
Glycitein	284.07	383.70	1.61	−1.13	0.04459	N
Pyrrolidonecarboxylic acid	130.05	513.40	1.58	−1.02	0.04517	P
L-Malic acid	133.01	44.40	1.53	2.01	0.04666	N

## Data Availability

The original contributions presented in the study are included in the article, further inquiries can be directed to the corresponding author.
